# Recommendations for Advancing Understanding of Eating Disorders in Neurodivergent People: A Commentary on Inal‐Kaleli et al. 2024 and Nimbley et al. 2024

**DOI:** 10.1002/eat.24380

**Published:** 2025-01-30

**Authors:** Moritz Herle, Virginia Carter Leno, Will Mandy

**Affiliations:** ^1^ Social, Genetic & Developmental Psychiatry Centre, Institute of Psychiatry, Psychology & Neuroscience King's College London London UK; ^2^ Centre for Brain and Cognitive Development, Birkbeck University of London UK; ^3^ Department of Clinical, Educational and Health Psychology UCL London UK

**Keywords:** ADHD, autism, disordered eating, lived experience, neurodiversity

## Abstract

Two recent reviews in the *International Journal of Eating Disorders* have highlighted the preponderance and unmet needs of neurodivergent people who experience disordered eating. In this commentary, we encourage researchers to engage with the bigger question of “What's Next?” and consider the type of research that is needed to shift the dial by lowering the incidence and persistence of disordered eating in neurodivergent people. As a starting point, we believe that future research must be guided by the needs and priorities of neurodivergent people with experience of eating disorders. Based on our own experience of community collaboration, we make specific recommendations for future research: (1) *Broadening the Scope*; such that we expand the focus beyond anorexia nervosa, and consider other manifestations of disordered eating, such as restriction, binge eating and emotional eating, and avoidant restrictive food intake disorder (ARFID), but also acknowledge the impact of other forms of neurodivergence beyond autism (e.g., ADHD), (2) *Identifying Causal Mechanisms*; moving beyond describing prevalence to studying why and how neurodevelopmental traits are associated with disordered eating (which in turn will inform new intervention design), and (3) *Adapting Existing Interventions*; understanding how current interventions can be adapted to meet the needs of neurodivergent individuals.

## 1 Summary of Findings

Recent research shines a light on the co‐occurrence of neurodevelopmental conditions, such as autism, with mental health problems, revealing that, compared to non‐autistic peers, autistic people experience elevated risk of a broad range of mental health conditions. Understanding the cause of this vulnerability and supporting neurodivergent people in need requires a multidisciplinary approach that brings expertise from what have historically been considered unrelated fields of research. In our case, we bring together expertise in eating behaviors, neurodevelopment, and clinical psychology, and we use our different perspectives to review and comment on the articles by Inal‐Kaleli et al. (2025) and Nimbley et al. (2025) in this issue of the *International Journal of Eating Disorders*. These valuable articles address two key questions: What is the prevalence of autism and autistic traits in patients with anorexia nervosa and How do autistic people experience existing treatment approaches for anorexia nervosa? Inal‐Kaleli et al. (2025) conducted a meta‐analysis to generate an updated estimate of the prevalence of autism and autistic traits among people with anorexia nervosa. Their meta‐analysis included 1172 people (mostly women) with anorexia nervosa and 2747 controls without anorexia nervosa. Their results indicated that people with anorexia nervosa had higher levels of autistic traits than the controls, and overall, about one‐third of people with anorexia nervosa in their study population met the criteria for an autism diagnosis using a gold‐standard research instrument. In the second paper by Nimbley et al. (2025), the authors took a mixed methods approach to systematically review 15 quantitative and 2 qualitative studies that investigated the experience and effectiveness of eating disorder treatment in autistic people. The review found that autistic people report worse experiences in eating disorder treatment than non‐autistic people, with longer treatment duration and increased risk of inpatient admission. Furthermore, the authors found higher rates of psychosocial difficulties in autistic people that persist after treatment, as well as concerns about the suitability of certain existing therapeutic interventions (e.g., group‐based approaches). Taken together, these reviews show that autistic people, and autistic women in particular, have a high need for effective eating disorder interventions; but that this need is not currently being adequately met.

Before detailing specific recommendations to move beyond this critical juncture and address this unmet need, a broad principle we wish to advocate for is that any future research should be guided by the priorities of people with lived experience, as well as the contributions from other stakeholders such as clinicians, friends and family members. This will ensure that future projects are in line with what people who have experienced disordered eating actually want from research and thus will have the greatest potential to improve lives and well‐being. We recently conducted a community consultation project with neurodivergent people with experience of disordered eating to identify their research priorities going forward (Keller et al. 2024). We took an iterative approach that included an online survey, analysis and topic refinement with a facilitated workshop to reach consensus on the top 10 priorities for the field. The topics could be conceptualized as falling into two overarching themes: improving clinical outcomes and identifying causal mechanisms for disordered eating in neurodivergent individuals. Many of our suggestions here are drawn from this project and chime with these broader themes. Based on our own experience of community collaboration, we make three specific recommendations for future research.

## 2 Broadening the Scope

We suggest a broadening of scope to both move beyond the predominant focus on anorexia nervosa to encompass the full range of disordered eating behaviors that neurodivergent people report experiencing—including binge eating, emotional eating, food avoidance—which is a core feature of ARFID. The narrow focus on restrictive eating may not capture the complexity of eating behaviors among neurodivergent individuals, potentially leading to gaps in treatment and support for those whose symptoms do not align with restrictive patterns. Standard treatment methods, often modeled around anorexia nervosa, may not address the needs of neurodivergent individuals experiencing other types of disordered eating. A wider focus will result in treatment models that reflect a broader, real‐world understanding of disordered eating. In this context, it is also important to differentiate between autistic eating behaviors that might be experienced as helpful for individuals and eating disorder symptoms, which can have negative impacts and people require clinical care to manage.

The second way in which we suggest the field could broaden its scope is to acknowledge the overlap of different forms of neurodivergence. To date, most research has focused on eating behaviors in autism, whereas it is known, for example, that individuals with ADHD, one of the most common neurodevelopmental conditions, also exhibit elevated rates of disordered eating behaviors, particularly binge eating (Kowalewska et al. 2024). This thinking should also include other neurodevelopmental conditions such as Tourette's syndrome, learning difficulties such as dyslexia, intellectual disability, along with genetic conditions such as Down's syndrome. Indeed, the current work by Inal‐Kaleli et al. acknowledges the complexity of interpreting the high estimated prevalence of autism given other psychiatric and neurodevelopmental conditions known to also co‐occur with anorexia nervosa. Expanding research on disordered eating beyond a sole focus on anorexia and autism will allow for a more inclusive perspective that respects a broader spectrum of neurodivergent people. Community‐driven studies that invite input from individuals with various neurodevelopmental conditions—such as ADHD, autism, and learning disabilities—can help understand the priorities of groups with different lived experiences and, thus, where research should focus next.

## 3 Identifying Causal Mechanisms

As alluded to in the paper by Nimbley et al. (2025), existing interventions for eating disorders may not be as effective for neurodivergent people. One reason behind this may be that the causal and maintaining factors for disordered eating are different in neurodivergent compared to neurotypical populations, such that key underlying mechanisms are autism‐ and/or ADHD‐specific. To be effective, interventions need to address the root causes of disordered eating, and thus, identifying said causal mechanisms should be a priority for future research.

Some have proposed that for individuals with ADHD, impulsivity and difficulties with emotional regulation might make binge eating or loss of control around food particularly challenging. In contrast, autistic individuals may avoid food due to sensory sensitivities, leading to restrictive eating patterns not always classified under traditional eating disorder frameworks. Emotional eating and binge eating might represent regulatory strategies used to cope with the stress of moving through a world that is not always suitable or affirmative for neurodivergent people and may be related to camouflaging or masking behaviors. If these hypotheses are borne out, they would have clear implications for clinical practice. For instance, therapeutic approaches targeting impulsivity might be more beneficial for individuals with ADHD and binge‐eating tendencies, whereas sensory‐focused interventions could help autistic individuals who struggle with specific food textures. Regulatory strategies, and a review of how emotionally distressing spaces or experiences may link to eating behaviors, could be beneficial to people from both autism, ADHD and wider neurodivergent communities. As it currently stands, we face large gaps in our understanding, which hinders the development of new interventions. Efforts to answer these questions could, as a first step, make use of existing longitudinal datasets, which have a measurement of relevant constructs, to begin to tease apart cause and effect over developmental time. However, it is likely that collecting high‐quality evidence of causal mechanisms will require new data collection to adequately capture constructs of interest. These novel data collection efforts require both resources and collaborative effort, bringing together multidisciplinary teams and people with lived experience to conduct relevant and robust science. When autism‐specific mechanisms are identified as causing and/or maintaining disordered eating, this will point to the need for the development of novel, targeted intervention strategies.

## 4 Adapting Existing Services for Neurodivergent People

In addition to improving our understanding of the mechanisms that underpin the heightened prevalence of disordered eating in people with neurodevelopmental conditions, it is also vital to understand how best to adapt existing services for neurodivergent clients. In their review, Nimbley et al. (2025) included quantitative assessment of eating disorder treatment outcomes in autistic people, but also reviewed qualitative data to understand the experience of eating disorder treatments. Their results suggest more needs to be done to understand not only what to target (as alluded to in the previous section on mechanisms), but also how support can be administered in a way that is accessible and affirming for neurodivergent people. Coproducing these services and holding the lived experience of people who will access these interventions at the center is key to providing accessible and effective support. One inspiring example is the Pathway for Eating disorders and Autism developed from Clinical Experience (PEACE), which provides clinical guidelines and treatment adaptations for autistic people with anorexia nervosa (Tchanturia et al. 2020). The program includes additional training for clinicians, a focus on specific sensory sensitivities commonly experienced by autistic people, and more individualized therapeutic interventions that move away from group‐based activities. Further, the PEACE pathway promotes psychoeducation and adapted materials, which can help clients communicate their needs with the clinical teams (e.g., communication passports) and prepare them for what they can expect on the hospital ward. It may be useful in the future to consider how principals and methods of the PEACE pathway can be disseminated and implemented in a wider range of settings accessed by neurodivergent people who want clinical support for disordered eating. This could encompass services for those with a wide range of eating challenges, across the lifespan, in community, outpatient and inpatient settings. A key element of this will be staff training to increase understanding of neurodiversity and neuroaffirmative practice.

## 5 Overall Summary

Coming back to our original theme of “What's Next?,” we hope we have provided some ideas for where research needs to focus moving forward. We suggest the field needs to take a broader approach, which acknowledges the complexity and diversity of both eating disorders and neurodevelopmental conditions, moves to identify causal mechanisms, and better understands how best to adapt existing interventions for neurodivergent people. Progress in these areas will provide the needed understanding to better support neurodivergent people who experience disordered eating. Both neurodevelopmental conditions and eating disorders are broad umbrellas; however, some conditions have been more prominent in recent years, and we hope that future research and practice will embrace the full spectrum and work collaboratively to improve people's lives. Most crucially, future research needs to work collaboratively with people with lived experience and integrate their experiences and priorities into research design, implementation, and dissemination. Only by working with community partners will the field be guided to ask and answer questions most important to those who have experienced disordered eating.

## Author Contributions

**Moritz Herle:** conceptualization, project administration, writing – original draft, writing – review and editing. **Virginia Carter Leno:** conceptualization, project administration, writing – original draft, writing – review and editing. **Will Mandy:** conceptualization, project administration, writing – original draft, writing – review and editing.

## Conflicts of Interest

The authors declare no conflicts of interest.
